# The Future of Education as a Creative Ecosystem: A Sociocultural Framework for the Development of Creativity

**DOI:** 10.3390/jintelligence10040099

**Published:** 2022-11-10

**Authors:** Felipe Zamana

**Affiliations:** Laboratoire de Psychologie et d’Ergonomie Appliquées, Université Paris Cité and Univ Gustave Eiffel, 92774 Boulogne-Billancourt, France; fezamana@gmail.com

**Keywords:** creativity, education, future, creative ecosystems

## Abstract

This article explores the social and educational impact post COVID-19 on education through the perspective of creativity. This is a reflective and forward-thinking piece of how creativity can transform the future of education. The article is structured into five parts. First, the opportunities and barriers that COVID-19 offers in preparing students for an uncertain future. Second, the recognition of the vital role of creativity in the future. Third, the article discusses the value of creativity in education. Fourth, the teachers’ role in stimulating creativity and how its practices can be encountered in 21st-century education is commented on. Fifth, the last section presents perspectives for the future of education in an uncertain and complex world, introduces the concept of creative ecosystems for education, and summarizes the key points related to the aspects to which education should devote its efforts in the coming years. The article questions if more creativity-focused education is possible in the future and promotes a deep reflection in this particular context for teachers and educational institutions about the topics that need more attention during this time of change.

## 1. Introduction

Humanity has faced major changes to adapt to as a global civilization due to the latest outbreak of the pandemic virus, COVID-19. These changes have spread across political, economic, and social spheres, having a direct and significant impact, especially in education.

Since World War II, there has been no common event capable of forcing countries to adopt urgent and drastic measures that push educational institutions around the world to suddenly use technological tools to create content and remote learning experiences for students (Arroio 2020; Kaur and Bhatt 2020).

However, even though most of the technological solutions adopted had already been available and had been familiar to us, changing the school environment from a physical classroom to a digital one was a painful change for both teachers and students, showing how much most of the educational systems are unprepared for the future.

However, COVID-19 brought a golden opportunity to reconsider what matters most in education. Creativity plays a decisive role in the significant changes occurring at the moment and in new emerging economies that depend upon these changes, but most schools and universities are not preparing students to be creative and innovative (Wagner 2012). As reported by the World Economic Forum (2018), technologies are enabling new ways of organizing how value is created, and, in this scenario, creativity is among the most critical capabilities to build the future.

Many educational institutions and teachers are researching, evaluating, and implementing various solutions and strategies to strengthen higher education. However, there is not enough focus on changes and methodologies to provide a more creative education. Although there are several research studies on learning and creative teaching strategies, limited research has investigated the relationships between creativity, curriculums, and learning ecosystems using a framework model.

## 2. Preparing the Students for an Uncertain Future

COVID-19 has become a real threat and has radically changed the world within merely 100 days since the first case (Kaur and Bhatt 2020). The virus has taught even those who had not experienced it so obviously before that human life is vulnerable, nature is unpredictable, and in such a sudden extreme situation, it is hard to trust most institutions since they have reacted in impulsive and random ways (Agnoletto and Queiroz 2020; Azorín 2020). This situation has made many people reconsider the need for a change which cares for both present and future generations, and education has an important role to play in such a change (Wolff 2020).

Nevertheless, the problems in the educational system are long known. Before COVID-19, school systems’ detachment from students’ needs was already an issue (Robinson 2017). For Illich, schools were first conceived with a highly organized factory structure, where students are “natural resources to be molded by the schools and fed into the industrial machine” (Illich 1972, p. 65). We still can see pieces of evidence of this structure today, as shown by the report, “Schools of the future: Defining new models of education for the Fourth Industrial Revolution” (World Economic Forum 2020a, p. 5):

Many education systems in developed and developing economies still rely heavily on passive forms of learning focused on direct instruction and memorization rather than interactive methods that promote the critical and individual thinking needed in today’s innovation-driven economy.

However, the pandemic was a critical moment for most educational institutions. For example, despite the students’ social issues, the school dropout rate, especially in higher education, skyrocketed compared to the pre-pandemic years (Nietzel 2021). For Azorín (2020), the central educational problems today also include, among others: the high rates of socioeconomic segregation, school dropouts, and academic failure; the poor culture of networking and collaboration; overcrowded classrooms that hinder quality education; and an obsolete curriculum. Besides these, most high school dropouts name boredom the number one reason they left (Diamandis 2018). From this point of view, teaching must be built to meet the needs of the new generation, but also, in addition to the maturity and experience of teachers and educational institutions, it must bring complementary ways to better prepare students for the future. Thus, this world is not just ours, so it is imperative for our youth to learn that social and collective awareness is indispensable (Usak et al. 2020).

The unprecedented experience of the pandemic showed how collective actions are fundamental to overcoming global challenges, and how every action counts (Arroio 2020). For Beck (1992) and Wolff (2020), humanity needs to be able to think systemically, seeking to anticipate the impact of its actions on multiple levels and contexts, and the ability to prevent these risks depends on access to knowledge and information.

In this sense, researchers are looking into new educational strategies on how we can give technology a better use to overcome former constraints and embrace new learning opportunities, such as the use of e-learning platforms (Wagner and Dintersmith 2016; Oranga and Matere 2022). Moreover, studies have shown that introducing students to future-readiness skills could offer them “[t]he ability to respond flexibly, make in-formed decisions, and adapt to rapid change” (Jalil et al. 2022, p. 1).

The mark of this time is uncertainty, more than ever. The risks in modern society are not distributed according to wealth or social position; they hit the entire society. The coronavirus crises have shown how new alliances of decision making can emerge, which means that the present time is the time to act and make a change.

In brief, there is no educational knowledge or skills that present a definitive solution to face a constantly changing educational or professional environment. This demands constant research and rethinking education to prepare students for the future, especially in a post-digital world. Therefore, teaching these students how to think creatively seems to be a good way to equip them with the capacity and potential to navigate uncertainty and change.

## 3. Expectations for the Future of Education

There is a unique opportunity to rethink what the future of education will look like. The situation caused by COVID-19 also surfaced the possibility to create a better education for all, focusing on the student’s well-being and needs (Fullan and Gallagher 2020). However, Azorín (2020) and Hargreaves (2020) question if this pedagogical renewal will arrive soon or if it will slowly go back to the same old way of schooling.

Today, creative performance does not seem to be a priority in schools, although the development of student creativity is crucial for economic, scientific, social, artistic, and cultural advancement (Amabile 2012; Cropley 2012; Garcês et al. 2016; Runco et al. 2016; Richardson and Mishra 2017). For Alencar, few students are capable of being inventive and original, and “it is necessary to prepare the student to solve problems that have not yet arisen—and this is only possible through stimulating creativity” (Alencar 2016, p. 18).

For example, with the emergence of new technologies such as artificial intelligence, more specifically machine learning and deep learning, the future of education can expect a blended model where machines will play a bigger role in schools (Mijwil et al. 2022). We are witnessing the rise of digital learning platforms such as Google Classroom and even YouTube, but also huge platforms like Coursera where companies and universities can offer training. According to Sushama et al. (2022), these platforms provide a type of service that saves time (e.g., no need to go physically to a classroom), reduces bureaucracy, and provides the learner with real-time feedback on the learning process.

The COVID-19 crisis also offers us an excellent opportunity to build the right motivation that directly influences characteristics such as curiosity, willingness to take risks, tolerance, dedication, energy, concentration, and fascination with the task, which are fundamental for creative thinking (Morais and Almeida 2019).

Morris et al. (2022) analyzed different global agencies’ reports in response to the educational challenges for the post-COVID-19 era, where most of them emphasized the need for a model or framework that can anticipate, optimize, and enhance the role of collaboration between the private sector and governments. Moreover, according to an analysis by McKinsey & Company (Craven et al. 2022):

- **Schools are the true fulcrum for the functioning of society.** Many solutions adopted were quick fixes that did not even focus on the real issue. Therefore, schools must be at the forefront of knowledge and teach students how to deal with uncertainty and complex problems. So, what should be the role of the school in building the future, and which are the “lessons learned” from the pandemic?
- **Work will never be the same.** The traditional work format was questioned when the way many employees worked was put in check. These changes brought up how deep the skill gap is and how urgently we need to upskill the workforce. Nevertheless, do the schools teach these new sets of skills to properly prepare the students for the future? How do our current educational practices really help in this process?
- **Government policy matters—but individual behavior sometimes matters more.** We cannot just sit and wait for help. Teachers, schools, and government must work together to implement the changes we need. What can we do to well prepare our students for the future?

Therefore, it is necessary that the educational systems be restructured around disciplines that involve more collaboration groups, improvisation, and creative processes, where the student can better respond to the needs and challenges of the current world. For this, a structure is needed that allows the possibility of reviewing and re-evaluating all situations and, if necessary, the courage to change things or keep them as they are.

## 4. Education as Creative Ecosystems

New emerging economies are creating and being fueled by major changes, such as the creative economy (see Howkins 2013), and so it is crucial to understand how education can prepare students for it and how creativity can be stimulated to aid them to adapt to these changes (Kadushin 2012; Simonton 2019). For example, good teachers know how important it is to have long-term planning for the student’s learning success; to achieve the main objective, communicating the content involves motivating and engaging each student in the teaching and learning process (Agnoletto and Queiroz 2020).

However, with the chaos caused by the COVID-19 pandemic, most educational institutions adopted digital technologies only to ensure that teachers have covered all the topics in the syllabus by the end of the academic year and to “save” the educational programs (Agnoletto and Queiroz 2020; Wan 2020). For Azorín (2020), remote learning is showing very clearly that a significant number of our teachers do not have adequate digital competencies, which leads the teachers to try to replicate the “classroom model” online, ignoring their different approaches. Besides that, the logic adopted is still “one size fits all”, ignoring that this “all” is represented by different individuals with their own pace, stories, needs, evolutions, and times.

The context of the pandemic highlights the recognition that education only makes sense if it is anchored in universal values such as human rights, empathy, and solidarity (Arroio 2020). The role of education has often been the rescuer, with a mission to change both individuals and society, but neither educators nor schools are free agents in society. With new technologies, the planet has become a learning space, and the student’s profile is entirely different from what it was in the past. In this sense, we can understand education as the integration of groups, social or cultural, where each member contributes to transforming it (Wagner 2012; Runco et al. 2016).

Education is part of an ecosystem; that is, it is made up of subunits—like buildings, classrooms, teachers, and students—and it may itself be the subunit of some broader collectives—such as communities, neighborhoods, and cities—and the dynamic interactions between them (Kauffman 1993; Harrington 2011). In this sense, education is about how people interact and grow together. So, if major characteristics that make us human are interpersonal relationships and the ability to create, creative ecosystems are human relationships directed towards creation—of knowledge, ideas, citizens, and societies (Morin 1999; Amabile 2012; Zamana 2021).

All societies share the same benevolent core principles, most of them essential for fostering creativity (Amabile 2012; Turner and Pennington 2015; Kauffman 2016; Christakis 2019). Creative ecosystems offer agility but integration on larger scales, emphasizing the skills of strategic empathy, collaborative leadership, and communication that rely on their members’ strength and creative capacity.

Creativity is present at different scales, from the individual to the social level; but independently of how it manifests, it needs some degree of recognition by the collective (Plucker et al. 2004; Glăveanu 2018; Glăveanu et al. 2019). So, in this perspective, creativity is never an individual act but a systemic interaction between the student and their sociocultural environment. For Glăveanu (2010), creativity cannot be detached from our historical and cultural contexts, especially when we became more aware of our social influences and started to emphasize creative collaboration and co-creation. For collaboration to happen, adaptation should be an indispensable criterion for creativity’s analysis, once the individual, as a member of the creative ecosystem, needs to be able to fully engage with its culture and environment (Cohen 2012; Runco 2017; Reeves 2019).

However, individuals can also create the conditions to shape the environment according to its needs and desires or abandon this environment to pursue another more favorable one for the development of their skills and interests. In this sense, learning is related to a culture of shared values and beliefs, which are built together (Fleith 2019). So, the environment should provide opportunities for individuals to develop their capacities but also recognize and encourage them during the process.

The development of students’ creative thinking enables them to have the necessary tools to seek knowledge and learn on their own in the future (Alquatahni 2016). The main objective of stimulating creativity in the classroom is to meet the demands of modern life, allowing the student to take advantage of their development opportunities (Amabile 2012; Alquatahni 2016; Morais and Almeida 2019). Therefore, education must continually renew itself and constantly look for new ways of teaching, focusing on how students learn and how they can appreciate their creativity, investing in the training of students who are able to fully enjoy their creative potential. For Alencar (2016, p. 7):

The awareness that it is necessary to invest in the training of individuals able to make full use of their creative potential has grown exponentially, and education institutions should prepare students for an uncertain future in a complex society marked by numerous challenges and demands.

By enriching the way students experience the world, they will be better prepared for the future, considering their cultural context and adapting to it (Cohen 2012; Runco 2017). Adapting to the context allows the individual to be tolerant of the world’s uncertainties or ambiguities, to accept not always having the answers, to be wrong, and to try new alternatives (Robinson 2017). In this case, educational institutions should provide students with a way to recognize their strategies and styles of working and thinking, as well as different forms of learning.

There is a need for something that facilitates the teaching of creativity and its learning methods, which are transversal to all areas and ages. Usually, we are taught to separate, compartmentalize, and isolate learning instead of making connections, and, consequently, our knowledge can become an “unintelligible puzzle lost between different disciplines” (Morin 1999, p. 17). This receptivity to new ideas and experimentation demands that we learn to consider and often look for ways to challenge current beliefs. The more we discover, the more we understand how incomplete our knowledge is, and with creative ecosystems it is possible to expand interest in the new, propose challenging goals, remain open to new experiences, and, above all, collaborate to build a better future together.

## 5. The Teachers’ Role in Stimulating Creativity

Much is said about stimulating the student’s creative potential, but the need to prepare the teacher for this development is forgotten. Like the student, the teacher also needs to study and prepare to be able to teach creatively, developing his teaching techniques and creativity, which will directly influence the quality of student learning (Mullet et al. 2016).

The COVID-19 situation provided most educators with the possibility to create a new way, or at least a different one, of teaching, which represents a chance to rescue the true value of education (Arroio 2020). The teacher should encourage openness but eliminate possible blocks, as the teacher has the vital role of intermediating the learning process through clues, guidance, and rectification. In this sense, to (re)think about education and the role of the teacher in the future to come, three main challenges need to be addressed:

**Less focus on outcomes and more focus on process.** The development of creative maturity involves both an external transformation of a specific field and an internal transformation of the self, which involves openness and willingness to change the current way of thinking for a new perspective (Alencar 2016; McCarthy and Blake 2017). However, the excessive focus on the outcomes leads to an overrated concern with practical knowledge. For example, most teachers have already heard students ask such questions as, “is it going to be on the test?” (Sharma and Scherrer 2014). In this logic, it is more about what the student decides to learn than what we want to teach (Pacheco 2014; Robinson 2017). Specific themes and approaches may seem more relevant for teachers, who are usually aware of the process’s importance, but are these themes and approaches relevant to the student?

Thus, the role of the teacher is taking a different path, as the students need more guidance through their learning process. A permissive educational environment can facilitate students’ creativity, but they must have the teacher’s attention and support to be aware of their potential (Runco et al. 2016). Through these interactions with the teacher, students internalize the problem-solving and learning processes, which will make them better prepared to deal with problems and challenges in the future (Hargreaves 2020). The development of creative maturity helps the students adapt to the environment, fit their plans and theories, and adjust to this new environment (Alencar 2016). Over time, as the teacher develops the student’s ability to generalize and transfer what they have learned, the teacher starts to encourage more than guide. When the students see meaning in what they are learning, it is easier to stimulate curiosity and encourage them to explore new perspectives, offering them the freedom to make their own decisions (Kaufman 2018).

**Less focus on the individual and more focus on the collective.** The World Economic Forum (2020b) listed social and emotional skills among the “most wanted” for the future of work, such as collaboration and teamwork. However, besides these skills not being taught at schools, students are penalized for asking questions, sharing their thoughts, and helping their peers in the classroom (Robinson 2017). What is more, we still hope that these same students become good citizens and participate actively in our society.

Moreover, most educational alternatives have proposed to converge toward the idea that individual needs are only met through specialization once we believe that our world is knowable, optimizable, and controllable, and this belief leads to overconfidence and dependence on specialists (Illich 1972; Kauffman 2016; Wolff 2020). Education urgently needs openness to individual choices, understanding how they impact the collective and, vice versa, teaching how we can build more meaningful relationships through solidarity, empathy, and respect (Arroio 2020).

For example, if promoted healthily, competition can favor teamwork and even facilitate understanding of various topics addressed in the classroom (Tang and Werner 2017; Morais and Almeida 2019). For this, education must take into account the students’ reality and experiences, creating spaces that favor sharing with a loose and low-supervised structure for learning (Pacheco 2014; Zamana 2021). Why not give the students the opportunity—and responsibility—to learn with each other?

**Less focus on the now (short term) and more on the future (long term).** If education is one of the pillars of any society, we will increasingly depend on decentralized and shared knowledge built by many hands (Kadushin 2012; Christakis 2019; Zamana 2021). Moreover, we should be cautious that the students do not become limited by our own views of the world. For example, the millennial generation, the latest to finish college and join the workforce, are considered the “burnout generation”, with high cases of depression and financial problems (Petersen 2020). So, it is hard not to wonder: what happened during their education years for them to have such a troublesome future?

Thus, there is no way to measure learning with tools focusing only on the immediate, specifically memorization models. From this point of view, standards and assessments need to be developed and not be limited to measuring whether students can complete a test, but if they have 21st-century skills such as communication, critical thinking, problem solving, entrepreneurship, collaboration, and, mainly, creativity. The classroom is just a device for learning to happen, but it is not always the best device. Education must stop being centered on traditional teaching in order to be centered on the relationship between individuals. As mentioned before, learning happens through collaboration, one of the most valued requirements for the new education (DaVia et al. 2018; Azorín 2020). The time has come for the educational systems to face the undeniable consequences of being out of date and provide the necessary changes for students to enjoy learning.

## 6. Conclusions

Creativity in education has become essential in modern society, and understanding its past not only allows us to understand its future but also to broaden our horizons and to see that our current situation is not constant and immutable since we have many more possibilities ahead of us than we imagine (Morin 1999; Harari 2017; Osmond-Johnson et al. 2020). In short, if there were ever an opportunity to draw on the talents and strengths of the collective capacity, that time is now.

To belong to that future, the individual must be creative and charismatic and able to recognize patterns and create meaning. If the students leave school without knowing how to create and innovate continually, they will be unprepared for society’s challenges. The development of this understanding is a task for the education of the future, with a fairer and more supportive educational system that can change lives. Otherwise, we will have missed out on this opportunity.

Creative ecosystems can represent a solution to educational and social challenges once local governments gradually embrace them (e.g., The Great Reset Initiative from the World Economic Forum). These purpose-driven ecosystems aim at solving significant social challenges and are also snowballing in importance, driving greater involvement of the public and not-for-profit sectors. Creative ecosystems can offer an agile and collaborative framework for a shift in education, providing the conditions for the development of social and emotional skills, such as leadership, strategic empathy, and communication.

Creative ecosystems will undoubtedly play a more significant role in the continuing growth of most societies. Engaging with creative ecosystems can provide education the freedom it needs to finally become a space for sharing knowledge, embracing change, fostering relationships, and building desirable futures (Turner and Pennington 2015; Bourgeois-Bougrine et al. 2020). Because of their unpredictability and spontaneity, creative ecosystems offer a greater idea yield from the assembled brains than traditional approaches. Hence, there are gaps for new research opportunities. Finally, with world society facing many new challenges (social, ecological, economic, etc.), perhaps creative ecosystems can help education in preparing students in this regard.

The pandemic situation due to COVID-19 is a wake-up call for education. Before this crisis, it was common to hear about the educational changes needed for the 21st century, but now we see how urgent these changes are. It is not the first time we have faced a pandemic situation, but it seems that we still cannot deal with unforeseen problems properly and act fast to solve them. The main reason is not that we are not capable of thinking creatively but that we are not taught to think that way; in fact, the worst thing you can do in school is to act creatively (Robinson 2017).

Maybe the entire idea of education needs a transformation. Educational institutions should make it possible to question the status quo, offer a safe space to share ideas, build trust and friendships, give up the need for control and stability to make room for experimentation, and emphasize empathy, collaboration, and communication. Changes, even radical ones, bring new beginnings.

Education needs to raise doubts and provoke the students to formulate good questions and not narrow down the student to the “right answer” because an unpredictable future is a “what if?” future. Therefore, we must prepare ourselves for the changes in the best way possible, and education should inspire the students of today and tomorrow to create new ways to make this world a better place for everyone.

## Data Availability

Not applicable.
